# Comparative analyses of the V4 and V9 regions of 18S rDNA for the extant eukaryotic community using the Illumina platform

**DOI:** 10.1038/s41598-020-63561-z

**Published:** 2020-04-16

**Authors:** Jaeho Choi, Jong Soo Park

**Affiliations:** 1 Department of Oceanography, School of Earth System Sciences, Kyungpook National University, Daegu, 41566 Republic of Korea; 2 Research Institute for Dok-do and Ulleung-do Island, Kyungpook National University, Daegu, 41566 Republic of Korea; 3 Kyungpook Institute of Oceanography, Kyungpook National University, Daegu, 41566 Republic of Korea

**Keywords:** Ecology, Environmental sciences

## Abstract

Illumina sequencing is a representative tool for understanding the massive diversity of microbial eukaryotes in natural ecosystems. Here, we investigated the eukaryotic community in a pond (salinity of 2–4) on Dokdo (island) in the East Sea, Korea, using Illumina sequencing with primer sets for the V4 and V9 regions of 18S rDNA from 2016 to 2018 for the first time. Totally, 1,413 operational taxonomic units (OTUs) and 915 OTUs were detected using the V9 and V4 primer sets, respectively. Taxonomic analyses of these OTUs revealed that although the V4 primer set failed to describe the extant diversity for some major sub-division groups, the V9 primer set represented their diversity. Moreover, the rare taxa with <1% of total reads were exclusively detected using V9 primer set. Hence, the diversity of the eukaryotic community can vary depending on the choice of primers. The Illumina sequencing data of the V9 region of 18S rDNA may be advantageous for estimating the richness of the eukaryotic community including a rare biosphere, whereas the simultaneous application of two biomarkers may be suitable for understanding the molecular phylogenetic relationships. We strongly recommend both biomarkers be used to assess the diversity and phylogenetic relationship within the eukaryotic community in natural samples.

## Introduction

The advent of next-generation sequencing (NGS) led to a substantial change in the previous knowledge about the microbial diversity in natural ecosystems^1,2^. NGS targeting the 18S rDNA is usually used to evaluate the diversity within all domains of life and provides large quantities of sequencing data for individual investigators. The 454 platform is hardly used anymore and a lot of other platforms are currently used far more widely. The Illumina platforms are representative tools for the investigation of the microbial community although this method can read a relatively short fragment of sequence (200 bp–500 bp) due to the technical limitations^3,4^. Illumina platform is a cost-effective tool per base (priced ~100 times lower than the 454 platform) and can now read longer sequences (200 bp–300 bp) than the initial platform^5,6^. Furthermore, the error rate of the Illumina platform is lower than that of the 454 platform^7^. However, considering the revolutionary application of the Illumina platform, our knowledge about the diversity and phylogenetic relationship of eukaryotes remains poor in field surveys such as those for brackish water^8^.

Several studies on the diversity of eukaryotes noted that the V1–V2, V3, V4, and V9 regions of 18S rDNA have been used for better understanding the massive diversity of microbial community^9–11^. The V4 (expected amplicon size, 270 bp–387 bp) and V9 (expected amplicon size, 96 bp–134 bp) regions are considered to be the popular for metabarcoding^12,13^. Based on an *in silico* analysis, the V9 region of 18S rDNA offers the advantage to reveal the extant diversity of eukaryotes, whereas the V4 region of 18S rDNA is commonly used for studying the phylogenetic relationship of eukaryotes^14^. Despite these advantages of both V4 and V9 regions of 18S rDNA, multiple primer sets have been employed rarely for environmental samples^15–17^. Furthermore, only a few specific eukaryotic groups (e.g., Chlorophyta, Trichomonads, Cercozoa, Radiolarians, and Ciliophora) have been elucidated for their diversity and phylogenetic relationship in environmental samples using NGS methods^10,18–21^. Subsequently, comparative analyses of the V4 and V9 regions have been uncharted in field surveys, and most important eukaryotic groups remain unexplored using the Illumina platform for both the V4 and V9 regions^22^. Further, most NGS studies had been focused mainly on the diversity of dominant eukaryotic groups rather than on the rare eukaryotic taxa that play a crucial role in the eukaryotic community in natural ecosystems^23,24^. Therefore, fundamental interrogations remain unanswered regarding the region of 18S rDNA that is appropriate to describe the diversity and phylogenetic relationship of the dominant or rare eukaryotic groups using the Illumina platform in field surveys, particularly of brackish water.

In the present study, we investigate the eukaryotic community in a brackish small pond on Dokdo (island) using the Illumina sequencing platform with the V4 and V9 regions of 18S rDNA from August 2016 to June 2018. The relative abundance of the major eukaryotes at the Class level in the original supergroups ‘Amoebozoa’, ‘Archaeplastida’, ‘Chromalveolata including Stramenopiles, Cryptista, Haptista, and Alveolata’, ‘Excavata’, ‘Opisthokonta’, and ‘Rhizaria’, represent inconsistent results based on the sequencing data of the V4 and V9 regions sequencing data of 18S rDNA. The V9 region can reveal most of the important eukaryotes or rare eukaryotic taxa, whereas the V4 region remains poorly covered in field samples. In the phylogenetic analyses of enormous eukaryotes, the V4 region has a much better resolution than the V9 region. Although the molecular detection of eukaryotes in the field samples is still far from complete, the assignment of environmental sequences depends on the choice of primer regions at the class (mostly) or higher level. Furthermore, the V4 region that is longer than the V9 region is appropriate to explain the previous evolutionary relationship of eukaryotes.

## Results

### Characteristic of the Mulgol pond in Dokdo (island)

**Figure 1:**
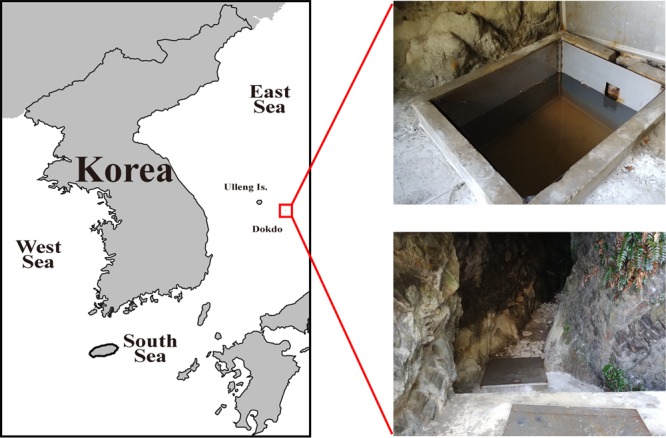
Study area and sampling site (the Mulgol pond) on Dokdo (red square box), Republic of Korea. Note that a map was created using the Mapping Toolbox in MATLAB (MATLAB R2019b). Is.: Island.

The water temperature of the Mulgol pond was between 14.5 °C and 17.6 °C in August 2016, September 2017, and April and June 2018. The salinities of the pond ranged from 1.49 to 2.70, indicating that this pond contained brackish water at the time of sampling. Light intensity of surface water was measured to be ~4.5 lux. However, this value could be much lower because of the metal lid blocking light source from the Mulgol pond (Fig. 1). The concentration of chlorophyll-*a* ranged from 0.07 to 0.50 µg L^−1^ during the whole study period.

### Overall Illumina sequencing data

A total of 780,706 V9 reads and 874,604 V4 reads were obtained by Illumina sequencing within a total of ~24 L of subsamples (6 L × 4 surveys in two years) of brackish water (Table 1). Sequence reads were filtered when the sequences had an ambiguous base, low quality (quality score offset of 33), chimera, and short reads (less than 36 bp) (Table 1). Total read count was 534,846 for the V9 region of 18S rDNA and 584,264 for the V4 region of 18S rDNA (Table 1). The length of the V9 sequence fragments ranged from 123 bp to 215 bp (average, 141 bp), whereas the length of the V4 sequence fragments was between 146 bp and 564 bp (average, 300 bp). These reads were assigned to OTUs at the same level of sequence identity. Individual sequences were clustered at 97% identity threshold. This study used a 97% identity threshold to cluster sequences to assign OTUs because of an agreement with several recent studies that used these primer sets^14,16,25^. With a 97% identity threshold, 1,632 V9 OTUs and 1,122 V4 OTUs were obtained (Table 1). After filtering OTUs assigned to prokaryotes, the unambiguous numbers of eukaryotic OTUs were 1,413 V9 region sequences and 915 V4 region sequences (Table 1).

**Table 1:** Summary of Illumina sequence data from the V4 and V9 regions.

Sequence description	V4	V9
Total bases	378,053,256	138,975,040
Read count	874,604	780,706
Filtered read count	584,264	534,846
Ambiguous	0	78
Low-quality	2,876	5
Chimera	20,040	25,204
Other (non-sequencing error)	267,424	220,573
OTUs (Total)	1,122	1,632
OTUs (Eukaryotic reads)	915	1,413

The ambiguous indicates filtered sequences with ambiguous base calls. The low-quality indicates filtered sequences with low-quality bases (Quality score offset 33). The term ‘other’ was defined as a non-sequencing error, which indicates query coverage and identity percentage with < 85%.

### Diversity analyses

**Figure 2:**
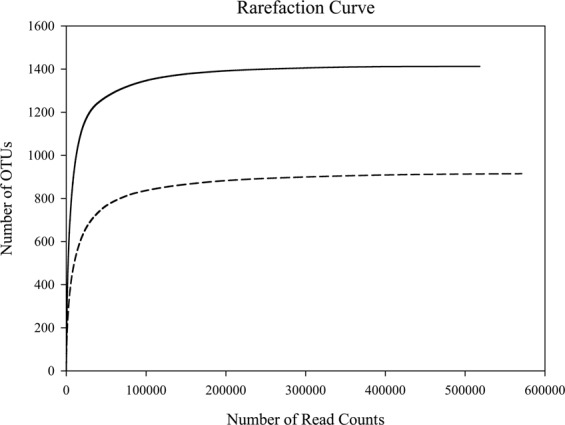
Diversity rarefaction curve of the V4 (dotted line) and V9 (solid line) operational taxonomic units (OTUs) with 97% identity threshold. The saturation of curve indicates that the diversity of OTUs in the present study was mostly covered by the V4 and V9 sequences.

Rarefaction analysis was conducted to determine whether OTUs in data has been sufficiently covered (Fig. 2). The saturation phase of the two rarefaction curve for the V4 and V9 regions of 18S rDNA indicated that the coverage of Illumina sequencing was sufficient during the whole study period. Eukaryotic OTUs were categorized into major sub-division groups (mostly class level) as reported by Adl *et al*.^26^. (see appendix 3. table 3.1). Of total reads, 57,997 V9 reads and 131,413 V4 reads could not be assigned to the class level, but these unclassified class reads could be successfully assigned to their supergroups at the highest rank level. Thus, 99.99% of total V9 data and 99.95% of total V4 data reads could be successfully assigned to their supergroups. The remnants of the unknown reads, which do not fall into a specific supergroup, were assigned to the ‘non-assigned’ group (Supplementary Table 1). Only a small fraction of total reads (0.01% of V9 data and 0.05% of V4 data) was characterized in this group.

**Figure 3:**
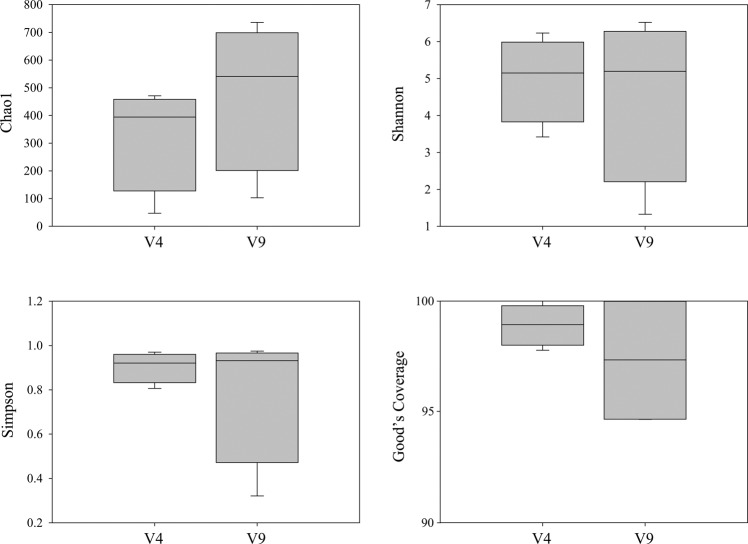
Alpha diversity indices for Chao1, Shannon diversity, inverse Simpson, and Good’s coverage in V4 and V9 region datasets. Note that whiskers above and below the box plot (interquartile range) represent the 90^th^ and 10^th^ percentiles. The lines within the boxes indicate the medians.

The Chao1 values ranged from 103 to 735.9 (average, 480.2) for the V9 region, and from 47 to 471 (average, 326.8) for the V4 region (Fig. 3). The observed OTUs were able to cover from 99.6% to 100% of the richness of the estimated species from Chao1 analysis. The Shannon diversity index represented the evenness of species that varied from 1.33 to 6.52 (average, 4.56) for the V9 region and from 3.42 to 6.23 (average, 4.99) for the V4 region (Fig. 3). The inverse Simpson index represents the probability of two randomly selected taxa belonging to the same species. In this study, the index varied from 0.32 to 0.97 (average, 0.79) for the V9 region and from 0.81 to 0.97 (average, 0.90) for the V4 region (Fig. 3). The Good’s coverage provides how well the sample represents the environment, and the coverages in this study was from 0.99 to 1 for both regions (Fig. 3). This illustrates that the generated reads from Illumina sequencing estimated the completeness of eukaryotic diversity in the samples.

### Comparative analyses of the V9 and V4 sequences data in field samples

**Figure 4:**
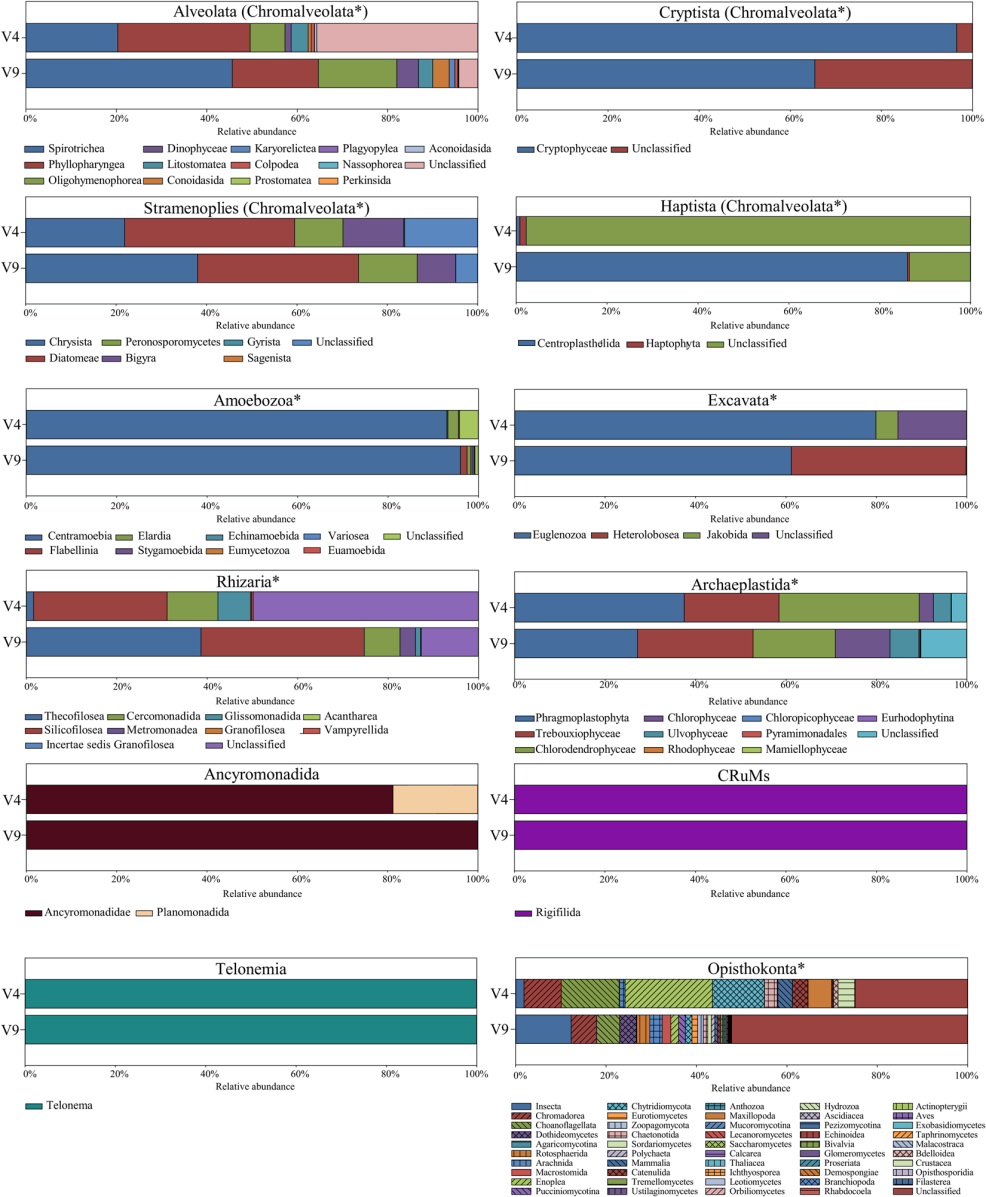
The relative fraction of major eukaryotic groups belonging to the original supergroups ‘Amoebozoa’, ‘Excavata’, ‘Rhizaria’, ‘Archaeplastida’, and ‘Opithokonta’ or the highest rank groups ‘Alveolata (Chromalveolata)’, ‘Cryptista (Chromalveolata)’, ‘Stramenopiles (Chromalveolata)’, ‘Haptista (Chromalveolata)’, ‘Ancyromonadida’, ‘CruMs’, and ‘Telonemia’ detected in the present V4 and V9 datasets based on read count (also see Supplementary Table 1). Asterisk (*) indicates the original supergroups in eukaryotes.

In this study, according to a recent classification and nomenclature of eukaryotes^26^, 94 and 70 different major sub-division groups were assigned from the V9 and V4 region of 18S rDNA sequences, respectively (total 102 separate groups, Supplementary Table 1). It appears that all sub-division groups described here belonging to the highest rank groups ‘Ancyromonadida’, ‘Cryptista’, ‘CRuMs’, ‘Haptista’, and ‘Telonemia’ could be recovered by both biomarkers V9 and V4 (Fig. 4 and Supplementary Table 1). Although a fraction of the major sub-division groups in total reads depended on the type of groups, except for the supergroup ‘Opisthokonta’, the major sub-division groups at the top rank in all supergroups were detected with both biomarkers. For instance, the sub-division group Thecofilosea in the supergroup ‘Rhizaria’ was 38.6% and 1.6% of the total reads in the V9 and V4 region sequences, respectively (Supplementary Table 1). Further, the sub-division group Centroplasthelida in the highest rank group ‘Haptista’ showed 86.1% and 0.8% of the total reads in the V9 and V4 region sequences, respectively (Supplementary Table 1). The unclassified subgroups showed usually a higher proportion of total reads at the supergroup or highest rank levels, regardless of the V9 and V4 regions. In the cases of ‘Alveolata’, ‘Amoebozoa’, ‘Excavata’, ‘Rhizaria’, ‘Stramenopiles’, and ‘Haptista’ all unclassified subgroups in the V4 region sequencing data appeared to a higher proportion than those in the V9 region sequencing data. On the bases of total V9 and V4 reads, the most considerable difference between the two biomarkers represented the supergroups ‘Excavata’ and ‘Amoebozoa’. Within the supergroup ‘Excavata’, the V9 region showed 87,300 reads in total, whereas the V4 region biomarker revealed only 329 reads (Supplementary Table 1). In the supergroup ‘Amoebozoa’, the relative numbers of the V9 and V4 region reads were 125,437 reads and 57,730 reads, respectively (Supplementary Table 1). Hence, it is hypothesized that the taxonomic affinities or reads intensely fluctuate depending on the choice of the primer sets.

Interestingly, the inventory of the major sub-division groups in the V9 region dataset did not correspond to that in the V4 region dataset. The major sub-division groups with almost <1% of total reads (rare subgroups) showed a substantial bias between V9 and V4 sequencing dataset, except ‘Opisthokonta’. The classes Karyorelictea, Prostomatea, and Nassophorea in Ciliophora and the family Perkinsida belonging to the highest rank group ‘Alveolata’ were not detected using the V4 sequencing data, whereas they were detected using the V9 sequencing data (Fig. 4 and Supplementary Table 1). Furthermore, the classes Echinamoebida, Eumycetozoa, and Euamoebida in the supergroup ‘Amoebozoa’ and Chloropicophyceae, Pyramimonadales, and Mamiellophyceae in the supergroup ‘Archaeplastida’ were not detected in the V4 sequencing data (Fig. 4 and Supplementary Table 1).

**Figure 5:**
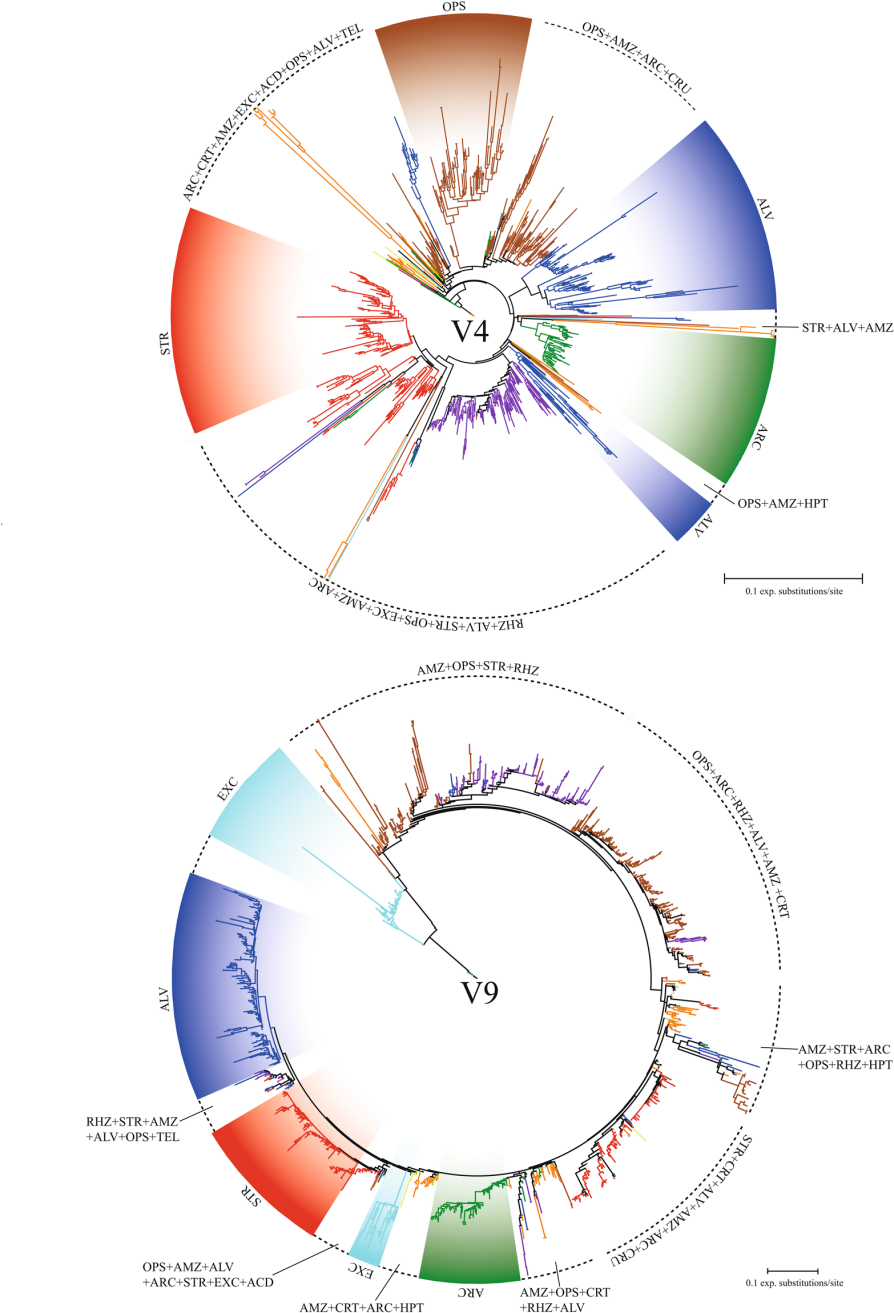
Unrooted maximum likelihood phylogenetic trees estimated from V4 (915 sequences total) and V9 (1,413 sequences total) region sequences of 18S rDNA using the Illumina MiSeq. Color of each node indicates a different supergroup or the highest rank group. OPS, Opisthokonta (brown); AMZ, Amoebozoa (orange); ALV, Alveolata (blue); STR, Stramenopiles (red); ARC, Archaeplastida (green); CRT, Cryptista (yellow); RHZ, Rhizaria (purple); EXC, Excavata (sky blue); HPT, Haptsita (light green); ACD, Ancyromonadida (gold); CRU, CRuMs (silver); and TEL, Telonemia (pink).

In most NGS studies, the V4 or V9 region sequences from NGS data have been placed into an alignment of almost full-length 18S rDNA sequences retrieved from the reference databases due to difficulty of alignment^27^. Very few attempts have been made for the phylogenetic inference using short V4 or V9 region sequences so far. In the present study, we examined phylogenetic inference amongst the supergroups or major sub-division groups reported by Adl *et al*.^26^ based on OTUs (Fig. 5). The molecular phylogenetic trees based on the V4 and V9 region sequences indicated that the eukaryotic supergroups formed a paraphyletic or polyphyletic group in our datasets (Fig. 5), confirming that current datasets have not resolved the previous phylogenetic relationships (i.e. a monophyletic group) at the highest-rank taxonomic group level. Furthermore, the length of the phylogenetic branch in the V4 region dataset was more varied than that in the V9 region dataset (e.g. supergroup ‘Amoebozoa’, Fig. 5). Thus, it seems that the V4 region might contain a more hypervariable region compared to the V9 region. Conversely, at the lower taxonomic level, some major sub-division groups including 2–27 OTUs represented a robust clade with >90% bootstrapping supports, which was depending on the dataset (Supplementary Table 1). In maximum likelihood analyses, a total 7 major sub-division groups formed a clade with 93–100% bootstrapping supports in the V9 region dataset (i.e. Echinamoebida, Heterolobosea, Granofilosea, Centroplasthelida, Peronosporomycetes, Phragmoplastophyta, and Telonema; Supplementary Table 1). In the V4 region dataset, a total of 7 major sub-division groups (Spirotrichea, Peronosporomycetes, Centroplasthelida, Enoplea, Chaetonotida, Pezizomycotina, and Rigifilida; Supplementary Table 1) formed a robust clade with 100% bootstrapping support. Classes Peronosporomycetes and Centroplasthelida showed a strong clade in both the V4 and V9 region datasets (Supplementary Table 1). Although the sub-division groups in the V9 region dataset showed more diversity than the V4 region dataset, most of the major sub-division groups could not reveal a previous monophyletic relationship in both the V4 and V9 region sequences using an Illumina platform.

## Discussion

We studied the comparative analyses of V4 and V9 regions of 18S rDNA, which are widely used for next-generation sequencing, in the field surveys to address the following questions: Do V4 and V9 region sequencing data provide similar taxonomic profiles in the dominant or rare eukaryotes? How different are eukaryotes at the high-rank taxonomic group level? Overall, we strongly recommend that the V4 and V9 regions are used together to investigate the diversity and taxonomic assignment of eukaryotes in field surveys^13,28,29^, and it is possible that some important eukaryotic groups may be unexplored or underestimated by the V4 or V9 region alone in the environmental samples, particularly for rare subgroups.

The two biomarkers V4 and V9 regions show a remarkable difference using the same Illumina platform within a total of approximately 24 L subsamples (6 L × 4 surveys in two years) of brackish water. On the bases of raw total bases and raw read counts, the numbers for the V4 region were relatively higher than those for the V9 region. However, eukaryotic OTUs for V9 region (i.e. 1,413 OTUs) are 20% more abundant than those for the V4 region (i.e. 915 OTUs) at 97% identity threshold. The determination of the identity threshold is one of the critical steps affecting the number of examined OTUs^21,23^. At the higher identity threshold, the estimation of diversity in eukaryotes is more conservative^13,20^. Surprisingly, Tragin *et al*.^21^ and Piredda *et al*.^8^ demonstrated that the fraction of the V9 region in total eukaryotic OTUs was 20% higher than that of the V4 region at 97% and 95% identity threshold, respectively, in the field samples (i.e. Atlantic Ocean, Pacific Ocean, and the Mediterranean Sea). Additionally, Maritz *et al*.^20^ reported that the V9 region OTUs (i.e. 1,444 OTUs) were 27% higher than the V4 region OTUs (i.e. 824 OTUs) from sewage samples in New York City at 98% identity threshold. This result suggests that the number of examined OTUs for V9 region are often 20% more likely to be larger than that for the V4 region, regardless of the identity threshold with 95% or higher and sampling locations^21^.

The sequencing error rates from ambiguous, low-quality, and chimera reads in the V4 region (2.7% of total read counts) are almost equivalent to those of the V9 region (3.2% of total read counts). Furthermore, prokaryotic OTUs (i.e. 207 OTUs of the total 1,122 OTUs) from the V4 region sequencing data are similar to those from the V9 region sequencing data (i.e. 219 OTUs of total 1,632 OTUs), implying that non-eukaryotic sequences are often detected in the V4 and V9 region datasets due to capability of the primer sets to amplify the small subunit rDNA of all three domains^30^ (i.e. archaea, bacteria, and eukaryotes). However, low coverage and low identity reads with <85% in the V4 region dataset was much higher than those in the V9 region dataset, whereas the chimera sequences in the V4 region dataset were slightly lower than those in the V9 region dataset. This suggests that the artifactual sequences from the V4 region sequencing data are more abundant, except for the chimera sequences. Previous studies revealed that the longer sequence, such as those of the V4 region, was susceptible to sequencing bias^21,31,32^. Although our results confirm those of the previous studies that the numbers of the artifactual sequences vary depending on the V4 or V9 region, a proportion of the sequencing error rates in total read counts from the V4 and V9 regions are similar to each other (see above). Thus, it is likely that the sequencing error rates are considered to weakly impact the estimation of eukaryotic diversity, irrespective of the amplicon regions.

The Chao1 and Good’s coverage indexes do not discriminate between V4 and V9 region datasets. Although the average values are not significantly different from each other (*t*-test, p = 0.752), the average Shannon diversity and inverse Simpson indexes for V4 region is slightly higher than those for the V9 region. Several previous studies reported that the Shannon diversity and inverse Simpson indexes for V4 region were lower than those for V9 region^20,21,33^. Conversely, Hirakata *et al*.^30^ reported that the Shannon diversity and inverse Simpson indexes for V4 region were higher than those for the V9 region, consistent with our results. Probably, this difference may be due to the fraction of different OTUs, intra-individual polymorphism, and/or different sampling locations^18,21,30,34^.

In the supergroups ‘Amoebozoa’, ‘Excavata’, and ‘Archaeplastida’ or highest rank groups ‘Cryptista’, ‘Ancyromonadida’, ‘CRuMs’, and ‘Telonema’, the most abundant sub-division groups in the V9 region dataset represent identical eukaryotes taxonomic profiles in the V4 region dataset. Other eukaryotic supergroups (i.e. ‘Rhizaria’ and ‘Opisthokonta’) or the highest rank groups (i.e. ‘Alveolata’, ‘Stramenopiles’, and ‘Haptista’) do not allow for the identical profiles in both the V4 and V9 region datasets. The second or third abundant sub-division groups in the V9 region taxonomic profiles displays the most abundant groups in the V4 region taxonomic profiles. Thus, according to the V4 or V9 region datasets, the order of the most abundant group in some eukaryotes taxonomic profiles can be altered, suggesting different affinity of the V4 or V9 region primer sets to the sub-division groups^8,32,33^. Further, it seems likely that a first- to third-ranked sub-division groups, which mostly occupied >50% of total cumulative read counts, are unchanged with respect to dominance in eukaryotes taxonomic profiles in both the V4 and V9 region datasets. Thus, it is unlikely that the taxonomic profiles in the predominant groups result from the primer bias^13^.

The V4 region may often provide the missing information on the diversity of some important eukaryotic groups in comparison to the V9 region^8,9,21^. Our result indicates that the V4 region fails to detect some important eukaryotic groups (e.g. Heterolobosea) and underestimates the diversity of the major eukaryotic groups (e.g. Thecofilosea and Centroplastithelida). In addition, most protists supergroups (i.e. ‘Chromalveolata’, ‘Amoebozoa’, ‘Excavata’, and ‘Rhizaria’) show high numbers of the unclassified subgroups in the V4 region dataset. Salonen *et al*.^33^ noted that the unclassified protistan subgroups in the V4 region dataset were much higher than those in the V9 region dataset, similar to our result. It is possible that the V4 region sequences may be deficient in the current database compared to the V9 region sequences^32^. Thus, prior to the completion of both the V4 and V9 region databases, the choice of primer set plays a crucial role in estimating the extant diversity of the target eukaryotes in field samples.

Due to the short NGS amplicons of the V4 and V9 regions, the molecular phylogenetic analyses shows that each supergroup represents a paraphyletic or polyphyletic group, indicating the lack of phylogenetic clustering at the high-rank taxonomic level. Adl *et al*.^26^ reported that Echinamoebida, Heterolobosea, Granofilosea, Centroplasthelida, Peronosporomycetes, Phragmoplastophyta, Telonema, Spirotrichea, Enoplea, Chaetonotida, Rigifilida, and Pezizomycotina also tend to fall into a monophyletic group, consistent with our result from the Illumina MiSeq. Interestingly, the monophyletic clustering of the major sub-division groups from the V4 region sequences (i.e. 7 groups of total 70 major sub-division groups, 100% bootstrapping support) is somewhat reliable than the V9 region sequences (i.e. 7 groups of total 94 major sub-division groups, 93–100% bootstrapping supports). This result suggests that most of the major sub-division groups do not reveal a monophyletic clade, and numbers of monophyletic clades in V4 region dataset are similar to the V9 region dataset. Because all supergroups or most major sub-division groups failed to examine the monophyletic relationships across eukaryotes, a reliable approach should be developed for assessing the eukaryotic relationships on the Illumina MiSeq.

Since NGS technologies have been introduced, rare eukaryotic taxa can be recently accessed for their ecological roles in natural ecosystems^21^. Rare eukaryotic taxa play a crucial role as seeds for species succession or blooming and can be flexible to environmental changes^23,24^. In this study, we also detected rare taxa with <1% of total reads (i.e. rare subgroups) using either the V4 or V9 regions. However, patterns of rare taxa are varied depending on the V4 or V9 regions. The rare taxa in the supergroups ‘Amoebozoa’, ‘Rhizaria’, and ‘Archaeplastida’ or highest rank group ‘Alveolata’ remained unexplored using the V4 region but were detected using the V9 region. Thus, it seems that the V9 region reveals much better resolution for the detection of rare taxa in most protistan supergroups than the V4 region. Furthermore, Heterolobosea was revealed to be the second most abundant group (38.6% of total read counts) in the supergroup ‘Excavata’ using the V9 region, but this group was not detected using the V4 region. Heterolobosea, including amoeba, amoeboflagellate, and flagellate forms, represents one of rare taxa in environmental surveys^17,30,35^, but were commonly isolated from freshwater, marine, soil, and extreme environments^36–46^. Pawlowski *et al*.^17^ reported that Heterolobosea comprises <1% of total reads using NGS technology with only the V9 region. Thus, the rare taxon Heterolobosea may be successfully detected using the V9 primer set, rather than the V4 primer set. However, our study is spatially limited for accessing the diversity of rare taxa in protists. Thus, further studies are needed at other locations or during other seasons.

In conclusion, the simultaneous application of V4 and V9 biomarkers in 18S rDNA is certainly advantageous for evaluating the diversity and phylogenetic relationship of the dominant or rare eukaryotic community in brackish water in comparison to the V4 or V9 region alone. Notably, the two biomarkers should complement each other for analysis of metabarcoding in the eukaryotic community. Thus, we strongly recommend the two biomarkers be widely used together in field surveys.

## Methods

### Sample collection

Surface water samples were collected from the Mulgol pond on Dokdo (island), Korea (37°14′22″N, 131°52′08″E) that is located in the East Sea/Sea of Japan, in August 2016, September 2017, and April and June 2018 (Fig. 1). Surface water samples were carefully taken with a sterile 1 L polycarbonate bottle to exclude any floating debris. Dokdo was designated as a Natural Monument and is maintained as a natural conservation district^47^. To maintain its natural ecosystem, only limited people are allowed to enter. Water in the Mulgol pond was historically used as drinking water for residents on Dokdo, but now it is no longer used for this purpose. This pond is reconstructed and now covered with a metal lid to protect from pollutants by a Korean government^48^. Dimensions of Mulgol are 1.4 m (width) × 1.2 m (length) × 1.7 m (depth). Because this pond has been covered with a metal lid, it has a limitation of light resources^45,48^ (Fig. 1). The light intensity was measured by light meter TES-1332A (TES Electrical Electronic Corp., Taipei, Taiwan). Additionally, because of ambient seawater and wastes from abundant seabirds, this pond contains a high concentration of nitrogen and a little dissolved salt^45,48^. The concentration of chlorophyll-*a* was measured by a standard protocol as described by Parsons *et al*.^49^, and water temperature and salinity were measured using a digital salinity/temperature meter (EUTECH Salt 6 + , Thermo Fisher Scientific, Republic of Korea).

### Environmental DNA Extraction

A total of 6 L of each water samples from the Mulgol were collected and filtered through 0.45 µm pore-sized Durapore membrane filters (Merck Millipore, Billerica, MA, USA) using a vacuum pump (model DOA-P704-AC, GAST, Benton Harbor, MI, USA) during the field periods. Probably, dissolved extracellular DNA passed through 0.45 µm pore-sized membrane filters^50^. These filters were stored in a 50 mL conical tube at −20 °C and taken to the laboratory for further experiments.

For environmental DNA extraction, 20% (w/v) lysozyme (final concentration, Sigma-Aldrich, St. Louis, MO, USA) were directly added to each conical tube after the filters were cut into several pieces. The tubes were then incubated at 37 °C for 30 min. Further, 0.5 mg mL^−1^ proteinase K (final concentration, Sigma-Aldrich) and 1% sodium dodecyl sulfate (final concentration, Bioneer, Daejeon, Korea) were added to the conical tube, and the tubes were incubated at 55 °C for 2 hrs. Nucleic acids were further purified using DNeasy Blood and Tissue Kit (Qiagen, Hilden, Germany) following the manufacturer’s instruction. The concentration of extracted DNA was measured with Quantus fluorometer (Promega, Madison, WI, USA). The final concentrations of extracted DNA ranged from 0.68 to 14.19 ng μL^−1^.

### Illumina sequencing

The V4 and V9 regions of 18S rDNA were used for the Illumina sequencing. Primers V4 forward (5′-CCAGCAGCCGCGGTAATTCC-3′) and V4 reverse (5′-ACTTTCGTTCTTGATTAA-3′) were used to target the V4 variable region^13^ of the 18S rDNA, and primers V9 forward (5′-CCCTGCCHTTTGTACACAC-3′) and V9 reverse (5′-CCTTCYGCAGGTTCACCTAC-3′) were used to target the V9 variable region^28^. Amplification conditions for V4 regions comprised initial denaturing step at 95 °C for 5 min, followed by 10 cycles of 94 °C for 30 s, 57 °C for 45 s, 72 °C for 1 min, and then, 15 cycles of 94 °C for 30 s, 47 °C for 45 s, 72 °C for 1 min, and ending at 72 °C for 10 min^29^. Reaction conditions for the V9 region comprised an initial denaturing step at 94 °C for 3 min. followed by 30 cycles of 94 °C for 30 s, 57 °C for 60 s, 72 °C for 90 s, and ending at 72 °C for 10 min^28^. Library size was confirmed by running on Agilent Technologies 2100 Bioanalyzer using a DNA 1000 chip (Aligent, Santa Clara, CA, USA). Library was quantified using a qPCR as described in the Illumina qPCR quantification protocol guide. A paired-end read was performed with the Illumina platform (i.e. Illumina MiSeq, Macrogen, Republic of Korea).

### Bioinformatic analysis and phylogenetic analysis

Paired-end reads were merged from Illumina sequencing with Fast Length Adjustment of SHort reads 1.2.11 program (FLASH)^51^. Sequences were trimmed and filtered and clustered by using CD-HIT-OTU software (v.0.0.1 for Illumina rRNA data)^52^. Through this process, short reads were filtered and long sequences were trimmed. Using CD-HIT-DUP, filtered sequences were clustered with 97% identity threshold and assigned to operational taxonomic units (OTUs). Also, chimeras were filtered out and extra-long tails were trimmed. Taxonomic composition for each sequence from phylum to species was generated using QIIME UCLUST^53^. Reference data were used with 18S rDNA data in the National Center for Biotechnology Information. The alpha diversity (i.e. Chao1, Shannon diversity, inverse Simpson, and Good’s coverage) analyses were conducted by QIIME pipeline^54^. Totally, 1,413 and 915 sequences obtained from the V9 and V4 region datasets, respectively, were aligned using MAFFT program version 7, and then were subsequently edited by eye^55^. The V4 and V9 region datasets retained only the 316 and 113 unambiguously aligned sites present in all partial sequences, respectively. Maximum likelihood trees were estimated using IQ tree on the version of IQ-TREE 1.6.12^56–58^. TIM3 + F + I + G4 and TIM2e + I + G4 models were selected through best-fit model test option (-m TEST) for V4 and V9 regions, respectively^56–58^. Ultrafast bootstrapping with 1,000 replications (-bb 1,000) was performed to estimate the branch support. The detailed command for this analysis was as follows: iqtree -s V4.fasta -m TEST -bb 1000 -nt AUTO, iqtree -s V9.fasta -m TEST -bb 1000 -nt AUTO. Analysis of *t*-test was performed using SPSS for Windows (version 25, SPSS Inc.).

According to the eukaryotic classification and nomenclature reported by Adl *et al*.^26,59,60^, the original supergroups in this study were conventionally assigned as ‘Amoebozoa’, ‘Archaeplastida’, ‘Chromalveolata’, ‘Excavata’, ‘Opisthokonta’, and ‘Rhizaria’. Furthermore, ‘Chromalveolata’ was divided into ‘Alveolata’, ‘Cryptista’, ‘Haptista’, and ‘Stramenopiles’ as the highest rank group. ‘Ancyromonadida’, ‘Telonemia’ and ‘CRuMs (i.e. Collodictyonidae, Rigifilida, and Mantamonas)’ were regarded as the other highest rank groups.

## Supplementary information

Supplementary information.

## Data Availability

The V4 (SRR10539012-SRR10539015) and V9 (SRR10539016-SRR10539019) sequence data were deposited in the NCBI Sequence Read Archive (SRA) under accession numbers between SRR10539012 and SRR10539019, and corresponding sample descriptions are accessible through BioProject PRJNA592034.
