# Supplementary material for: Comparative analyses of the V4 and V9 regions of 18S rDNA for the extant eukaryotic community using the Illumina platform
Source: Sci Rep. 2020 Apr 16;10:6519. doi: 10.1038/s41598-020-63561-z (PMC7162856; doi:10.1038/s41598-020-63561-z)
Supplement: Supplementary file 1 — Supplementary information. [file 41598_2020_63561_MOESM1_ESM.pdf]

TITLE PAGE FOR SUPPLEMENTARY TABLE 1

Comparative analyses of the V4 and V9 regions of 18S rDNA for the extant eukaryotic community using the Illumina platform

Jaeho Choi[1], Jong Soo Park[1,2,3*]

[1]Department of Oceanography, School of Earth System Sciences, Kyungpook National University, Daegu 41566, Republic of Korea

[2]Research Institute for Dok-do and Ulleung-do Island, Kyungpook National University, Daegu 41566, Republic of Korea

[3]Kyungpook Institute of Oceanography, Kyungpook National University, Daegu 41566, Republic of Korea

[*] Correspondence to: Jong Soo Park, Department of Oceanography, School of Earth System Sciences, Kyungpook National University, Daegu 41566, Republic of Korea; email: jongsoopark@knu.ac.kr; tel.: +82 53 950 5391; fax: +82 53 950 5397

Supplementary Table 1. The taxonomic profiles of eukaryotes based on the sequences of the V4 and V9 regions. Out of 102 major sub-division groups, the V9 region could detect 94 major sub-division groups that were 92% of the total eukaryotic major sub-division groups, whereas the V4 region could detect 70 major sub-division groups that were only 69% of the total eukaryotic major sub-division groups. Only values of 90% or above are shown for statistical bootstrapping values with 1000 replicates. † indicates the number of OTUs in each major sub-division groups. * indicates the original classification of supergroups or highest rank groups. P: phylum level, C: class level, O: order level, F: family level.

Alveolata (Chromalveolata*)

Major sub-division	Abundance		Statistical support (†)	
	V4	V9	V4	V9
Spirotrichea (C)	20.3%	45.6%	100%(27)	
Phyllopharyngea (C)	29.3%	19.1%		
Oligohymenophorea (C)	7.7%	17.3%		
Dinophyceae (C)	1.4%	4.8%		
Litostomatea (C)	3.8%	3.1%		
Conoidasida (C)	0.7%	3.6%		
Karyorelictea (C)		1.3%		
Colpodea (C)	0.7%	0.7%		
Prostomatea (P)		0.1%		
Nassophorea (C)		<0.1%		
Perkinsida (F)		<0.1%		
Aconoidasida (C)	0.5%			
Unclassified Alveolata	35.7%	4.3%		
**Total Reads**	115,337	69,776		

Cryptista (Chromalveolata*)

Major sub-division	Abundance		Statistical support (†)	
	V4	V9	V4	V9
Cryptophyceae (O)	96.5%	65.4%		
Unclassified Cryptophyceae	4.5%	34.6%		
**Total Reads**	637	1,173		

Stramenopiles (Chromalveolata*)

Major sub-division	Abundance		Statistical support (†)	
	V4	V9	V4	V9
Chrysista (C)	21.9%	38.0%		
Diatomeae (C)	37.6%	35.6%		
Peronosporomycetes (C)	10.7%	13.0%	100%(20)	97%(21)
Bigyra (C)	13.4%	8.5%		
Gyrista (C)	0.2%	<0.1%		
Sagenista (C)	<0.1%			
Unclassified Stramenopiles	16.2%	4.9%		
**Total Reads**	165,326	102,141		

Haptista (Chromalveolata*)

Major sub-division	Abundance		Statistical support (†)	
	V4	V9	V4	V9
Centroplasthelida (C)	0.8%	86.1%	100%(2)	99%(10)
Haptophyta (C)	1.4%	0.4%		
Unclassified Haptista	97.8%	13.5%		
**Total Reads**	2,489	3,677		

Amoebozoa*

Major sub-division	Abundance		Statistical support (†)	
	V4	V9	V4	V9
Centramoebia (C)	93.0%	96.0%		
Flabellinia (C)	0.2%	1.5%		
Elardia (C)	2.3%	0.8%		
Stygamoebida (C)	0.2%	0.5%		
Echinamoebida (C)		0.3%		100%(4)
Eumycetozoa (C)		0.1%		
Variosea (C)	<0.1%	<0.1%		
Euamoebida (C)		<0.1%		
Unclassified Amoebozoa	4.2%	0.7%		
**Total Reads**	57,730	125,437		

Excavata*

Major sub-division	Abundance		Statistical support (†)	
	V4	V9	V4	V9
Euglenozoa (P)	79.9%	61.2%		
Heterolobosea (P)		38.6%		94%(22)
Jakobida (P)	4.9%	0.1%		
Unclassified Excavata	15.2%	0.1%		
**Total Reads**	329	87,300		

Rhizaria*

Major sub-division	Abundance		Statistical support (†)	
	V4	V9	V4	V9
Thecofilosea (C)	1.6%	38.6%		
Silicofilosea (C)	29.5%	36.2%		
Cercomonadida (F)	11.3%	7.9%		
Metromonadea (F)		3.4%		
Glissomonadida (O)	7.2%	1.1%		
Granofilosea (O)	0.2%	<0.1%		93%(3)
Incertae sedis Granofilosea (O)	<0.1%	<0.1%		
Vampyrellida (O)	0.3%	<0.1%		
Unclassified Rhizaria	49.9%	12.7%		
**Total Reads**	40,994	26,794		

Archaeplastida*

Major sub-division	Abundance		Statistical support (†)	
	V4	V9	V4	V9
Phragmoplastophyta (C)	37.5%	27.1%		95%(25)
Trebouxiophyceae (C)	21.0%	25.6%		
Chlorodendrophyceae (C)	31.0%	18.2%		
Chlorophyceae (C)	3.2%	12.1%		
Ulvophyceae (C)	3.9%	6.4%		
Rhodophyceae (P)	<0.1%	0.2%		
Chloropicophyceae (C)		0.1%		
Pyramimonadales (C)		0.1%		
Mamiellophyceae (C)		<0.1%		
Eurhodophytina (C)	0.1%			
Unclassified Archaeplastida	3.4%	10.2%		
**Total Reads**	25,862	17,522		

Opisthokonta*

Major sub-division	Abundance		Statistical support (†)		Major sub-division	Abundance		Statistical support (†)	
	V4	V9	V4	V9		V4	V9	V4	V9
Insecta (C)	3.1%	15.1%			Thaliacea (C)		0.1%		
Chromadorea (C)	8.2%	5.6%			Ichthyosporea (C)	0.2%	0.1%		
Choanoflagellata (C)	12.8%	5.1%			Leotiomycetes (C)		0.1%		
Dothideomycetes (C)	0.1%	3.7%			Orbiliomycetes (C)		0.1%		
Agaricomycotina (C)	<0.1%	0.1%			Hydrozoa (C)		0.1%		
Rotosphaerida (O)		2.9%			Ascidiacea (C)		0.1%		
Macrostomida (O)	<0.1%	1.9%			Pezizomycotina (C)	<0.1%	<0.1%	100%(2)	
Enoplea (C)	19.3%	1.7%	100%(3)		Echinoidea (C)	<0.1%	<0.1%		
Pucciniomycotina (C)		1.5%			Bivalvia (C)	<0.1%	<0.1%		
Chytridiomycota (C)	11.5%	1.5%			Glomeromycetes (C)	<0.1%	<0.1%		
Eurotiomycetes (C)		1.3%			Proseriata (O)		<0.1%		
Zoopagomycotina (P)	<0.1%	1.3%			Demospongiae (C)		<0.1%		
Chaetonotida (O)	2.8%	1.0%	100%(3)		Branchiopoda (C)		<0.1%		
Sordariomycetes (C)	<0.1%	0.8%			Rhabdocoela (O)		<0.1%		
Polychaeta (C)	0.1%	0.8%			Actinopterygii (C)	<0.1%	<0.1%		
Mammalia (C)	3.2%	0.7%			Aves (C)		<0.1%		
Catenulida (P)	3.4%	0.6%			Exobasidiomycetes (C)		<0.1%		
Tremellomycetes (C)		0.4%			Taphrinomycetes (C)		<0.1%		
Ustilaginomycetes (C)	0.1%	0.3%			Malacostraca (C)		<0.1%		
Anthozoa (C)		0.3%			Bdelloidea (C)	1.0%			
Maxillopoda (C)	5.2%	0.3%			Crustacea (C)	3.7%			
Mucoromycotina (P)	<0.1%	0.2%			Opisthosporidia (O)	0.1%			
Lecanoromycetes (C)		0.2%			Filasterea (C)	<0.1%			
Saccharomycetes (C)	0.2%	0.1%			Unclassified Opisthokonta	24.9%	52.2%		
Calcarea (C)		0.1%							
**Total Reads**						148,505	82,207		

Ancyromonadida

Major sub-division	Abundance		Statistical support (†)	
	V4	V9	V4	V9
Ancyromonadidae (F)	81.2%	100.0%		
Planomonadidae (F)	18.8%			
**Total Reads**	85	9		

CRuMs

Major sub-division	Abundance		Statistical support (†)	
	V4	V9	V4	V9
Rigifilida (F)	100%	100%	100%(2)	
**Total Reads**	36	6		

Telonemia

Major sub-division	Abundance		Statistical support (†)	
	V4	V9	V4	V9
Telonema (C)	100%	100%		100%(2)
**Total Reads**	265	77		

Non-assigned

Major sub-division	Abundance		Statistical support (†)	
	V4	V4	V4	V9
Non-assigned	100%	100%		
**Total Reads**	276	37		
